# Development and application of a standardized teaching assessment system for digital automated oral implantation planning skills: a randomized controlled pilot trial

**DOI:** 10.3389/froh.2026.1892776

**Published:** 2026-09-16

**Authors:** Shi Li, Kegui Hou, Yi Zhang, Wenting Wang, Zichuan Liu, Hongli Zhang, Zhaofeng Ma

**Affiliations:** 1 Department of Stomatology, Seventh Medical Center of Chinese PLA General Hospital, Beijing, China; 2 Department of Stomatology, Beijing Shunyi District Hospital, Shunyi, Beijing, China

**Keywords:** dental education, dental implantology, digital planning, implant placement training, implantology education

## Abstract

**Objectives:**

The objective of this study is to develop and preliminarily evaluate a digital automated standardized teaching evaluation system for implant placement training as a pilot study. The purpose of this system is twofold: first, to improve the standardization of professional teaching in implantology education, and second, to improve the efficiency of professional teaching in implantology education.

**Methods:**

This investigation was conceived and executed as a randomized controlled pilot trial. A digital automated standardized teaching evaluation system for implant placement training was developed. A total of 40 students enrolled in an oral medicine course who lacked prior experience with dental implant surgery were randomly assigned to either group. The experimental group was instructed to utilize the aforementioned system for the purposes of training, while the control group received traditional free-hand model training. A comparative analysis of the teaching experience of the two groups was conducted through the administration of questionnaires and the use of scales. The difference in teaching efficiency between the two groups was compared by conducting a statistical analysis of the time spent by teachers on grading. Dental implant placement accuracy was verified by analyzing planned and actual implant positions after a fixed number of teaching hours.

**Results:**

A comparative analysis revealed that students utilizing the system demonstrated marked enhancements in teaching efficacy and skill mastery when compared to the traditional teaching group. The efficacy of this system is evidenced by the significant reduction in the time allocated by teachers to assignment evaluation and the concomitant enhancement of teaching efficiency and student satisfaction.

**Conclusions:**

The present pilot trial offers preliminary evidence that the system enhances the efficiency of standardized preclinical dental implant training in short-term simulated settings. It is imperative that further research be conducted in order to substantiate the long-term.

## 1 Introduction

Dental implantation has emerged as the prevailing treatment modality for tooth loss, with its application in clinical practice showing a marked increase in recent years (1). However, due to its strong practicality requirements, most dental students cannot truly master it even at graduation or in the early stages of their careers (2). Conventional pedagogical methods frequently offer students limited opportunities to engage in practical operations, often presenting challenges in handling real-world cases. This limitation can impede the development of interdisciplinary thinking and adaptability in students (3–5). In model training environments that provide operational practice, students’ feedback during operations is largely dependent on teachers’ on-site observation and experience-based judgment. The evaluation criteria are inconsistent, which makes it difficult to achieve personalized guidance and precise teaching. Furthermore, educators frequently find themselves obliged to utilize numerous software applications to assess the post-implantation effects, a process that consumes a substantial amount of time and hinders students from receiving prompt feedback (6). These shortcomings have the potential to compromise the fairness and consistency of teaching evaluations and to impede the timeliness and scientific nature of teaching feedback. It is noteworthy that the current cohort of dental students is predominantly composed of Generation Z digital native learners, who are accustomed to acquiring knowledge through immersive, interactive digital approaches (7). A comprehensive digital teaching framework that incorporates standardized skill training, real-time procedural assessment, and immediate individualized feedback is well-aligned with their cognitive patterns and learning habits, constituting a fundamental requirement for the modernization of dental implantation education. Furthermore, minor errors in implant angle, depth, and position control can significantly affect clinical outcomes in dental implant placement, thus necessitating the introduction of higher precision and real-time correction suggestion evaluation methods (8). As digital automation technology continues to evolve within the domain of medical education, the development of automated, standardized, and personalized teaching systems has emerged as a pivotal avenue for medical education reform (9). The present study developed a digital automated standardized teaching evaluation system for implant placement training, with the objective of exploring its application value in improving teaching efficiency, enhancing students’ operational accuracy, and realizing timely learning feedback.

## 2 Materials and methods

The present manuscript has been prepared in accordance with the CONSORT 2025 Statement and its associated checklist, which is available in the Supplementary Material 1 (10). In accordance with the guidelines established by the International Committee of Medical Journal Editors (ICMJE), this study is designated as an educational pilot trial. Its primary objective is to assess the efficacy of the educational intervention and the outcomes of the training skills among the trainees. The evaluation does not encompass the assessment of clinical health outcomes in patients. Consequently, it is exempt from the requirements for trial registration. The complete study protocol is available from the corresponding author upon reasonable request.

### 2.1 Ethical considerations, randomization and blinding

The study protocol was reviewed and approved by the Ethics Committee of Beijing Shunyi District Hospital (protocol code 2026-K-012, approved on January 13, 2026). The recruitment period occurred from March 2026 to April 2026. Prior to enrollment, all participants were informed about the study's objectives, procedures, and potential risks. They were also explicitly notified of their unconditional, penalty-free right to withdraw from the study at any time. Their decision to withdraw would not affect their academic performance or course assessment results. Prior to participation, all subjects provided written informed consent. The collection and management of data adhered to stringent confidentiality standards, ensuring the complete anonymization of participant information. The present study was conducted in strict accordance with the ethical principles of the World Medical Association Declaration of Helsinki and institutional regulations governing research involving human participants.

Participants were randomly allocated to the two study groups at a 1:1 ratio using a computer-generated randomization sequence. The allocation concealment process was facilitated by the use of sequentially numbered, opaque, sealed envelopes. These envelopes were prepared by an independent researcher who was not involved in the recruitment of participants, the delivery of the intervention, or the assessment of outcomes.

Given the distinct operational differences between digital automated interactive training and traditional model-based teaching, it was not possible to blind participants or outcome assessors to group allocation. In order to minimize potential assessment bias, the system's built-in point cloud registration algorithm was used to automatically calculate all primary outcome metrics rather than manually score them. This ensured objective, standardized, and consistent measurement across all participants. Furthermore, statistical analysts were unaware of group assignment during the processes of data processing and analysis to mitigate potential bias.

### 2.2 Sample size

A formal sample size power calculation was not necessary for this study, as it was designed as a randomized pilot and feasibility trial (11). In accordance with the established sample sizes from analogous published studies on dental implant skills training (12, 13), a target sample of 40 participants (20 per study arm) was deemed appropriate.

### 2.3 Development of the digital automated standardized teaching evaluation system

The system was developed as a standardized intervention and assessment tool for this randomized controlled pilot trial of implant placement training. The aim of the trial was to target the core limitations of conventional implant training, which include inconsistent evaluation criteria, delayed feedback, and low teaching efficiency. The system was optimized based on a pre-existing dental implant planning platform [Yake Wisdom (Beijing) Technology Co., Ltd.]. The system comprises four core functional modules aligned with the study's outcome measures (Figure 1).

**Figure 1:**
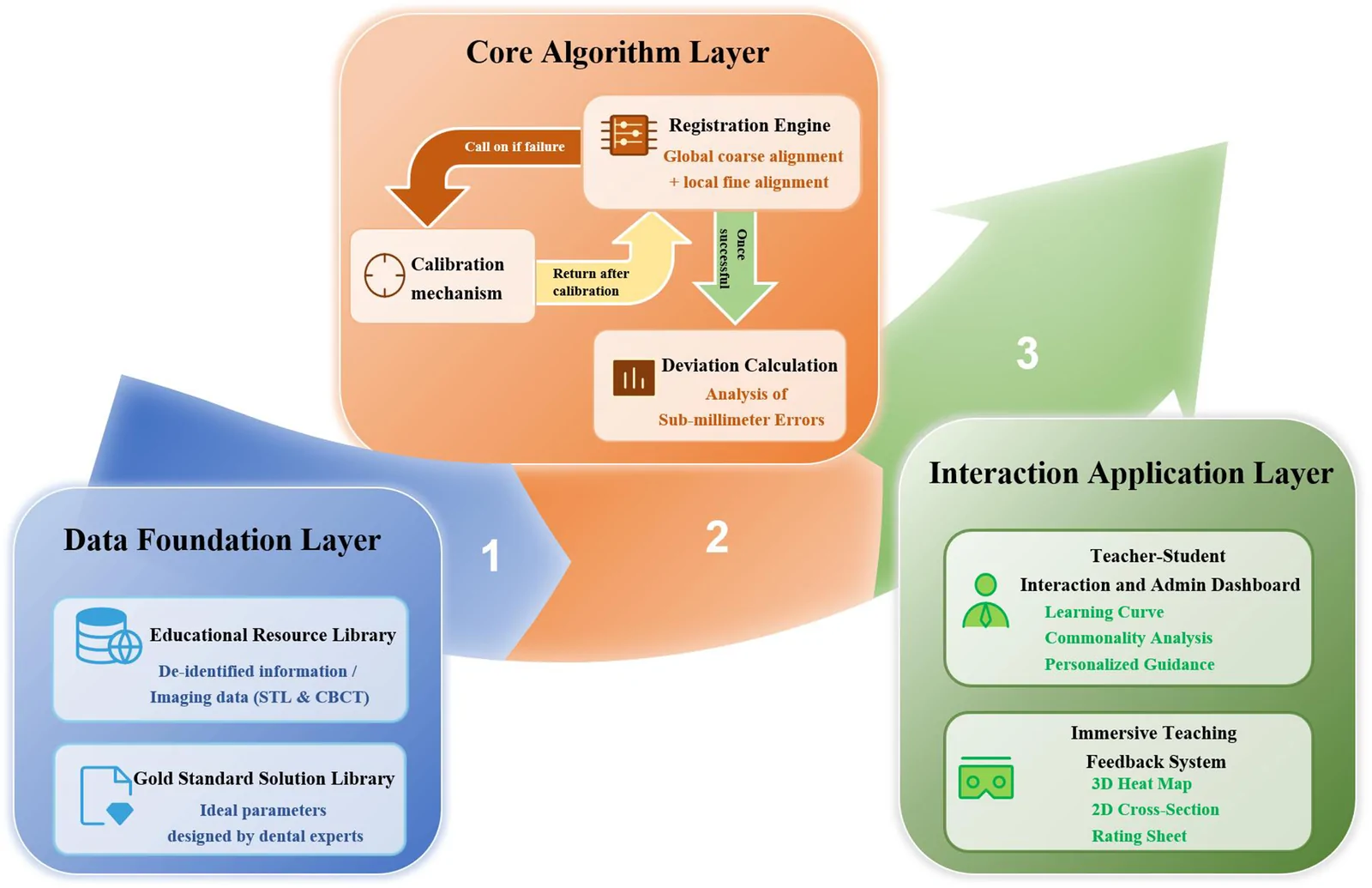
Schematic illustration of the design logic of the implant placement training evaluation system.

The modular structure of the system is illustrated in Figure 2.

**Figure 2:**
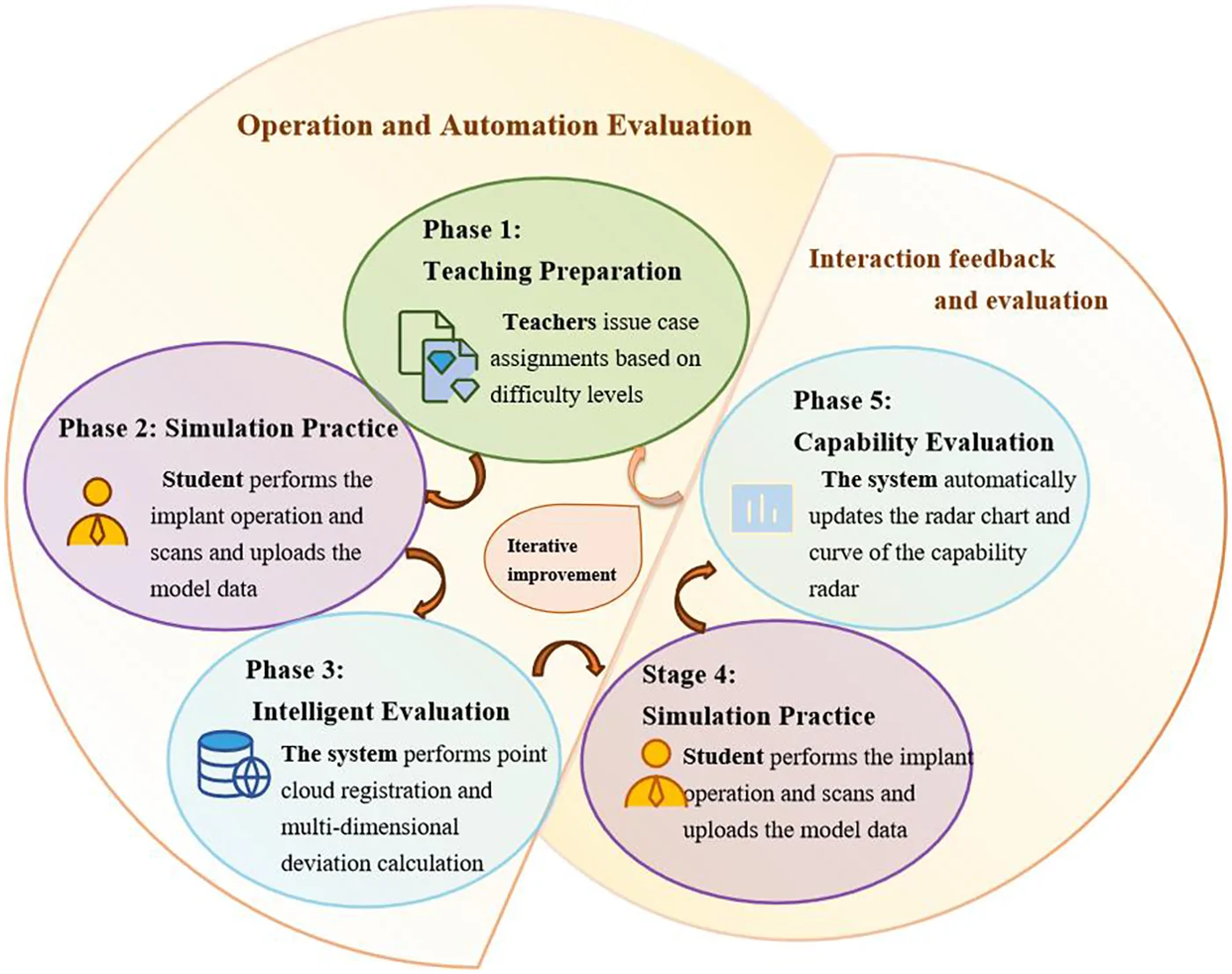
Flowchart of the process for using the implant placement training evaluation system.

#### 2.3.1 Teaching case library module

This module comprises a hierarchically managed case library with unified expert-endorsed gold standards. Each case contains desensitized clinical data, preoperative imaging, and a sub-millimeter-accuracy digital dental implant plan that has been developed by a minimum of two senior implant specialists. Cases are stratified by difficulty to ensure personalized, graded learning, and this approach guarantees standardized training across both study arms. This method also eliminates confounding from inconsistent training content. The clinical imaging data utilized to establish the standard case library is obtained retrospectively, undergoes complete de-identification, and is anonymized, ensuring the complete removal of any personally identifiable information. The utilization of these data for educational research purposes was formally endorsed by the institutional ethics committee, with a waiver of individual informed consent, in strict accordance with the principles established in the Declaration of Helsinki.

#### 2.3.2 Automated implant accuracy assessment module

The module employs the iterative closest point (ICP) point-cloud registration method to quantify three-dimensional (3D) neck, apical, and angular discrepancies between the planned and virtual implant positions. The DentalNavi v3.0 (Yake Wisdom) software was employed for all automated calculations. As indicated by the findings of the relevant *in vitro* experiments of this software, submillimeter precision was achieved via ICP matching (14). The 3Shape Trios3 scanner was utilized as the instrument for digitization. Previous clinical studies had validated the scanner's low median surface error (15), thereby ensuring the reliability of the raw scan data for subsequent deviation analysis. These automated discrepancy metrics were employed as objective primary outcome measures of participants’ implant placement performance.

#### 2.3.3 Interactive feedback module

The system produces an immediate visual feedback report following the submission of a task. This report contains a 3D deviation heatmap, cross-sectional anatomical comparisons, and a quantifiable scoring table that can be traced. The system provides real-time, visualized feedback, thereby addressing the delayed and ambiguous feedback issue inherent in traditional model training.

#### 2.3.4 Teaching management backend

The instructor backend facilitates the allocation of batch tasks, the automation of grading processes, the analysis of errors at the class level, the tracking of individual progress, and the provision of personalized guidance. The objective of the scale is twofold: first, to measure the secondary outcome (instructor grading time, i.e., teaching efficiency), and second, to support standardized management of the entire teaching intervention.

### 2.4 Research participants

In the present study, a total of 40 students without surgical experience studying dental implantology in our hospital were selected and randomly divided into two groups: a traditional training group and a digital automated system training group, with 20 students in each group. The random grouping results were generated by computer software. Prior to the commencement of the training, the grouping results were maintained in a state of confidentiality.

#### 2.4.1 Inclusion criteria

a. Students who are majoring in stomatology; b. No previous experience in any real or model-based implant placement operations; c. Passed the basic spatial perception ability test to ensure the comparability of the baseline levels of both groups [the score of Vandenberg-Kuse Mental Rotation Test ≥10 points (16)]; d. Proficient in basic computer operation: weekly computer usage time ≥1 hour, with basic experience in 3D imaging software, and self-rated overall computer proficiency ≥2 on a 5-point scale; e. Fully understand the research content and voluntarily sign a written informed consent form.

#### 2.4.2 Exclusion criteria

a. Individuals who have participated in any form of digital implant design or simulation training will be excluded from the study. b. Participants with a Vandenberg-Kuse Mental Rotation Test score of less than 10 will be excluded from the study, as this indicates low spatial cognitive ability. c. Individuals who disagree with the content of this study or do not sign the informed consent form will be excluded from the study.

### 2.5 Research content

The establishment of a replicable and standardized *in vitro* simulation operation and evaluation system is of paramount importance. The production of a series of standardized resin dental models of the missing right lower first molar (46 site) will be accomplished through the utilization of high-precision 3D printing technology. For one benchmark model, the acquisition of benchmark data is to be conducted using a high-precision intraoral scanner (e.g., 3Shape Trios3) and CBCT equipment. Subsequently, at least two associate chief physicians or above with more than 10 years of clinical experience independently carry out digital implantation scheme design in the DentalNavi software of Yake Smart Oral Surgery Planning. After exhaustive collective discussion, a final consensus is reached, and this scheme is established as the sole, unchangeable “gold standard” for evaluating all students’ operations. The result is then fully recorded in the teaching case library of the software.

#### 2.5.1 Group teaching and operational practice

The medical students participating in the experiment were randomly divided into two groups: the experimental group and the control group. This division was accomplished through the utilization of computer software. The experimental group utilizes the digital automated system developed in this study for practice and evaluation, thereby acquiring immediate learning experience; the control group adheres to the traditional teaching model in its entirety, with senior teachers conducting manual evaluation and providing feedback.

#### 2.5.2 Outcome measures

Outcome measures were defined *a priori*, with one primary outcome and two secondary outcomes, to comprehensively evaluate the efficacy of the digital automated teaching intervention. All baseline measurements of implant placement accuracy were strictly completed before randomization and initiation of the allocated training intervention, to confirm baseline homogeneity between groups and avoid intervention-related bias.

##### 2.5.2.1 Primary outcome: implant placement accuracy

The operational accuracy of the participants was assessed immediately following the completion of the full teaching course, which consisted of three 45-minute sessions delivered within a single week. The primary outcome metrics included 3D deviation of the implant neck (mm), 3D deviation of the implant apex (mm), and angular deviation of the implant axis (°), calculated by comparing each participant's dental implant placement against the expert-defined gold standard. All deviation data were exported directly from the system backend, with measurements recorded to two decimal places.

##### 2.5.2.2 Secondary outcomes

Teaching evaluation efficiency: The evaluation of the grading time was conducted by three experienced implant instructors who were not involved in the execution of the procedure. In order to assess potential clustering effects across instructors, inter-instructor reliability for grading time was calculated, showing good consistency and no significant clustering effect (Supplementary Material 2). Student subjective satisfaction and educational experience: Assessed using two validated anonymous questionnaires:

The System Usability Scale (SUS): A validated 10-item tool for digital system usability. Items use a 5-point Likert scale, with raw scores converted to a 0–100 standardized score (higher scores = better usability). It was used to assess the teaching system (17).
The Dundee Ready Education Environment Measure (DREEM): A validated 50-item scale covering five educational environment subdomains. Each item is scored 0–4; total scores range 0–200 (higher scores = more positive learning experience). It evaluated students’ overall educational perceptions (18). The validated Chinese versions of the above two scales were adopted in this study (19, 20), both of which exhibited satisfactory internal consistency in the present sample, as shown in Supplementary Material 2.

Comprehensive statistical analysis of these primary and secondary outcomes was performed to scientifically verify the application value of the digital automated teaching scheme in implantology education.

### 2.6 Statistical analysis

All randomized participants were included in the intention-to-treat (ITT) analysis set as the primary analysis population. No participants were lost to follow-up during the trial, and no missing outcome data were observed; complete-case analysis was therefore applied for all statistical comparisons. All statistical analyses were performed using SPSS version 26.0. A two-tailed *α* level of 0.05 was set as the threshold for statistical significance.

Continuous variables are presented as mean ± standard deviation (SD), while categorical variables are expressed as frequencies. The primary outcomes were analyzed using analysis of covariance (ANCOVA) (21), with baseline values (first practice session) of each indicator entered as covariates. Prior to ANCOVA, all underlying statistical assumptions were formally verified and satisfied, the results of statistical analysis have been supplemented in Supplementary Material 3. Secondary outcomes were compared using independent-samples t-tests.

For all analyses, exact *p*-values, test statistics (t or F), degrees of freedom (df), effect sizes (Cohen’s d for t-tests, partial *η*^2^ for ANCOVA), and 95% confidence intervals (95% CI) for mean differences are reported.

Given multiple accuracy outcome metrics and multiple questionnaire subscales, the Holm–Bonferroni method was applied to adjust *p*-values for multiple comparisons within each outcome domain. Exact *p*-values, test statistics, degrees of freedom, effect sizes and 95% confidence intervals are reported for all comparisons. *P*-values smaller than 0.001 are presented as <.001.

## 3 Results

### 3.1 The research team developed educational software for evaluating dental implants

The workflow is shown in Figure 3; specific steps are provided in Supplementary Material 4.

**Figure 3:**
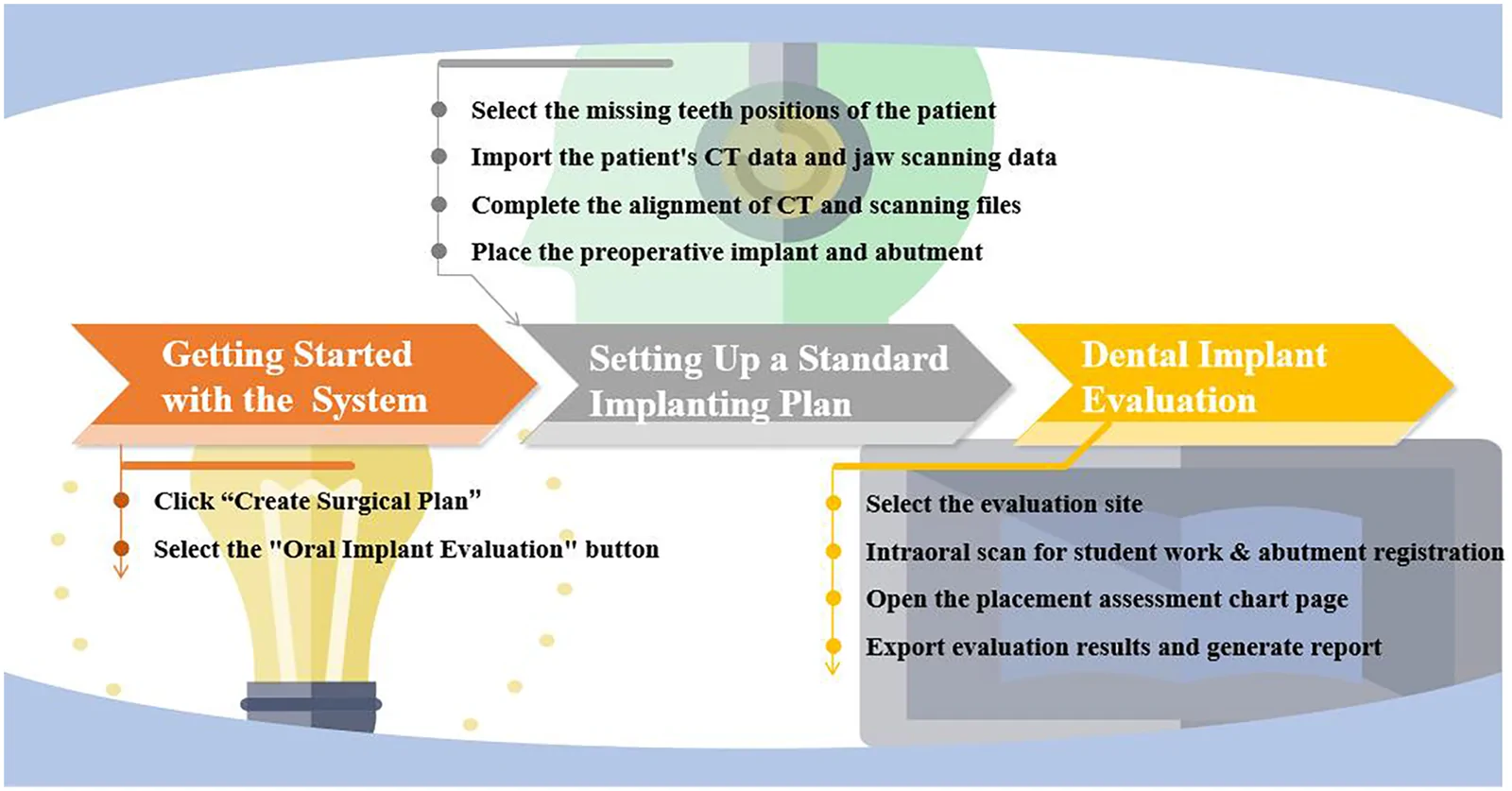
Flowchart for the dental implant teaching system.

### 3.2 Participants

As demonstrated in Figure 4 and Table 1, a total of 46 participants were initially screened for this study. Six participants were excluded from the study due to failure to meet the inclusion criteria or due to their decision to withdraw from the study before formal enrollment. The final sample comprised 40 participants who met the specified criteria and successfully completed the trial, which were subsequently divided into two groups of 20 subjects each. In the experimental group, the male-to-female ratio was 11:9, with a mean age of 21.1 ± 1.6 years; in the control group, the male-to-female ratio was 10:10, with a mean age of 21.3 ± 1.5 years. Prior to their involvement in the study, none of the students in either group had received any systematic training related to oral implant surgery. No adverse events or harms were reported during this non-invasive, simulation-based educational trial.

**Figure 4:**
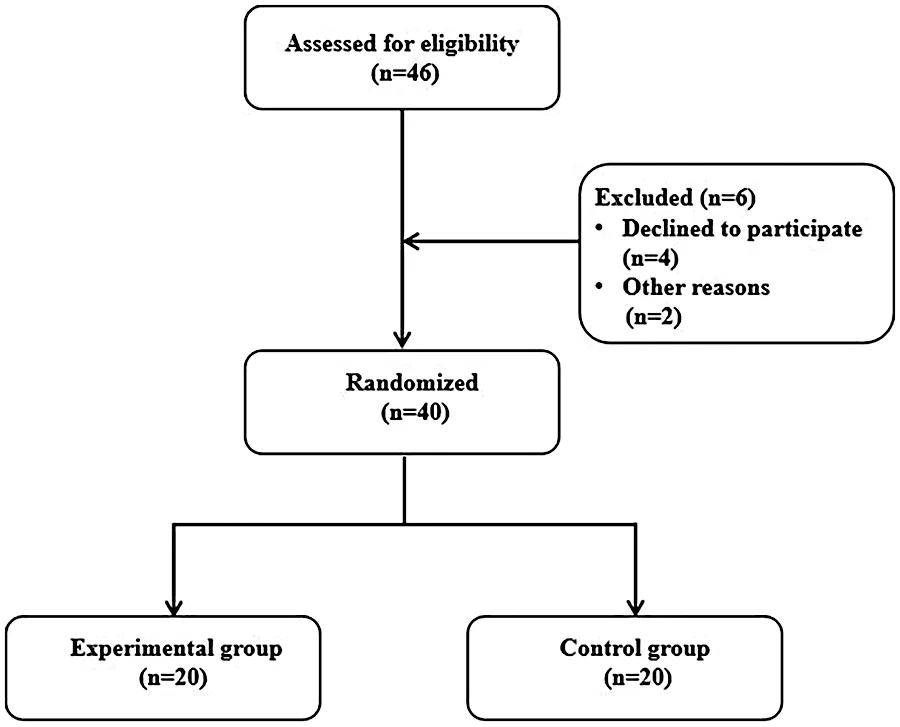
Participant flow.

**Table 1:** Baseline characteristics of participants.

	Number（N）	Gender		Age（years） (Mean ± SD)	Duration of implant course training (hours)
		Male	Female		
Experimental group	20	11	9	21.10 ± 1.60	0
Control group	20	10	10	21.30 ± 1.50	0
Total	40	21	19	21.20 ± 1.53	0

*Baseline comparisons: age, t = −0.41, *p* = 0.686; gender, *χ*^2^ = 0.10, *p* = 0.752. The two groups were well balanced at baseline.

### 3.3 Comparative end points or outcomes

As illustrated in Table 2 (including Parts A and B), **Part A** summarizes raw pre-training baseline and post-training implantation deviation data, while **Part B** presents analysis of covariance (ANCOVA) results adjusted for baseline performance for all primary implantation accuracy endpoints. Subsequent to the Holm–Bonferroni correction, all three deviation indicators exhibited statistically significant intergroup disparities (adjusted *p* < 0.001). The experimental group demonstrated significantly reduced adjusted mean deviations, accompanied by substantial partial *η*^2^ effect sizes that were observed across all three outcome metrics.

**Table 2:** Primary outcomes: between-group comparison of dental implant placement accuracy (ANCOVA adjusted for baseline values).

Part A. Descriptive statistics (Mean ± SD)						
Outcome Measure	Exp Baseline	Con Baseline	*P* Baseline	Exp post-training	Con post-training	*P* post-training
3D Deviation at the Implant Neck (mm)	2.85 ± 0.32	2.94 ± 0.35	0.403*	1.13 ± 0.45	2.04 ± 0.48	**<0**.**001**
3D Deviation at the Apical Region (mm)	3.37 ± 0.36	3.28 ± 0.39	0.453*	1.48 ± 0.54	2.43 ± 0.56	**<0**.**001**
Implant axial angle deviation (°)	5.59 ± 0.46	5.73 ± 0.49	0.354*	2.79 ± 0.85	4.47 ± 0.91	**<0**.**001**

Part B. ANCOVA between-group test results								
Outcome Measure	Exp Adj Mean	Con Adj Mean	Adj Mean Diff (95% CI)	F	df	p	*P* (Holm-Bonferroni)	Partial *η*^2^
3D Deviation at the Implant Neck (mm)	1.15	2.02	−0.87 [−1.16, −0.58]	37.19	(1, 37)	**<0** **.** **001**	**<0** **.** **001**	0.501
3D Deviation at the Apical Region (mm)	1.45	2.45	−1.00 [−1.34, −0.66]	35.94	(1, 37)	**<0** **.** **001**	**<0** **.** **001**	0.493
Implant axial angle deviation (°)	2.83	4.44	−1.61 [−2.16, −1.06]	34.88	(1, 37)	**<0** **.** **001**	**<0** **.** **001**	0.485

*No significant between−group differences were observed for all baseline accuracy indicators (all *p* > 0.05). Covariate: baseline scores from the first practice session. *P*-values were adjusted using the Holm–Bonferroni method for multiple comparisons across the three accuracy outcomes.

The bold values *p* < 0.001 represents significant difference between the two groups.

As demonstrated in Table 3, a series of independent-samples t-tests have been utilized to compare secondary outcomes. A substantial discrepancy was identified among the three indicators between the groups (*p* < 0.001). The experimental group exhibited a significant reduction in grading time, higher SUS usability scores, and higher total DREEM educational environment scores compared to the control group. The SUS and DREEM comparisons yielded substantial effect sizes (Cohen's d > 0.8). Table 4 presents the individual evaluation time ranges. The experimental group exhibited a marked reduction in evaluation time, with no overlap of individual values between the two cohorts.

**Table 3:** Secondary outcomes: between-group comparison.

Outcome Measure	Exp (Mean ± SD)	Con (Mean ± SD)	Mean Diff (95% CI)	t	df	p	Cohen's d
Evaluation Time (min)	3.50 ± 0.95	28.50 ± 4.20	−25.00 [−27.00, −23.00]	−25.96	38	**<0**.**001**	−8.21
SUS Total Score	81.25 ± 6.66	71.25 ± 8.56	10.00 [5.09, 14.91]	4.12	38	**<0**.**001**	1.30
DREEM Total Score	155.50 ± 9.47	130.30 ± 11.64	25.20 [18.41, 32.00]	7.51	38	**<0**.**001**	2.38

* The Levene test for Evaluation time indicated variance heterogeneity (*p* < .001), and Welch's correction was applied (df = 20.9); for SUS and DREEM, the variance was homogeneous, with df = 38.

**Table 4:** Range of individual operation time by group.

	Minimum time (min)	Maximum time (min)
Exp Time	1.87	5.28
Con Time	21.34	36.33

*No overlapping individual operation time values were detected between the two groups.

## 4 Discussion

The process of acquiring proficiency in dental implantology is often encumbered by several factors. Among these challenges are the use of subjective manual evaluation, delayed feedback, inconsistent teaching criteria, and an onerous faculty workload (22). The prevailing focus of contemporary digital training instruments is on preoperative implant planning. Despite the documented benefits of virtual reality/augmented reality (VR/AR) simulators in enhancing anatomical spatial awareness, their widespread adoption is constrained by several factors. These include the substantial costs associated with hardware acquisition, the paucity of diverse case scenarios, and the absence of customized quantitative feedback mechanisms (23, 24). To address these gaps, this study developed an integrated digital teaching and assessment system with four core modules. The system adopts combined coarse-to-fine point cloud registration to achieve sub-millimeter evaluation accuracy.

ANCOVA results adjusted for baseline performance revealed significantly smaller 3D positional and angular deviations in the experimental group, with large effect sizes (partial *η*^2^ > 0.48). This finding is consistent with prior research indicating that visualizing digital feedback enhances the precision of dental operations (25). By reducing the assessment duration from approximately 30 minutes to less than 5 minutes, the automated scoring module effectively addresses the significant time expenditure associated with manual evaluation (26).

Secondary outcomes revealed that the experimental group exhibited significantly higher SUS and DREEM scores, indicating enhanced system usability and an augmented educational perception. This finding aligns with the extant literature on digital dental education research (27). Distinct from static traditional assessment, the system generates interactive 3D deviation heatmaps and sectional comparisons to deliver instant error feedback, thus solving the problem of delayed guidance in conventional training (28). The longitudinal operation data storage and learning curve tracking capabilities facilitate the implementation of targeted, stratified tutoring, thereby establishing a comprehensive digital teaching loop. Additionally, the system facilitates the continuous manual updating of expert gold-standard case libraries, thereby enhancing the objectivity and standardization of teaching evaluations. Additionally, it facilitates remote guidance via online platforms, thereby enabling cross-regional teaching interactions and providing a viable pathway for talent development in economically underdeveloped and remote areas.

The development of this digital automated standardized teaching evaluation system for dental implant skills provides a novel auxiliary tool for preclinical implant skill training. This platform facilitates the unification of evaluation benchmarks and the acceleration of teachers’ grading efficiency. However, it is imperative to acknowledge the limitations of the present study and the system itself.

Firstly, the system depends on expert consensus for the calibration of measurement parameters and the formulation of gold-standard evaluation criteria for individual clinical cases. Subjective bias cannot be fully eliminated during expert criterion formulation, and large-scale multi-expert datasets and unified industrial assessment specifications are still required to reduce human interference. Secondly, the objective of this pilot educational trial was to compare teaching efficacy rather than to conduct independent metrological validation of the measurement software. Despite the validation of the ICP registration algorithm and DentalNavi measurement workflow in previously published *in vitro* experiments (14, 15), a dedicated repeated-measurement reliability test with standardized resin models will be conducted in subsequent technical research to further quantify the full measurement performance of this automated assessment system. Thirdly, the present trial adopted a single-center pilot design with a small sample of 40 dental students, a short-term standardized training session, and no long-term follow-up observation. The collection of clinical implant outcome data was not undertaken to ascertain whether enhanced preclinical performance is indicative of superior intraoperative clinical operation proficiency. A fourth possibility is that participants exhibited a novelty effect when using the newly developed system. This could introduce performance bias and overestimate the actual training effect of the platform (29). Fifthly, the generalizability of the training system remains unconfirmed. The potential for adapting the assessment and training framework for utilization in other implant-associated surgical procedures, including bone grafting and maxillary sinus floor elevation, remains to be elucidated. Sixth, the integration of auxiliary technologies, such as virtual reality and intraoperative surgical navigation, remains in its nascent stages. Further targeted research is necessary to expand the application of these technologies in the domain of preclinical education.

## 5 Conclusion

The present study developed a digital automated standardized evaluation system for preclinical implant skill training. In this preliminary investigation, the system demonstrated encouraging potential in enhancing the standardization of evaluation criteria and reducing instructors’ grading burden. The study also elicited favorable SUS and DREEM feedback. Substantial additional research is necessary to draw robust conclusions about its broad educational utility. This additional research should include technical refinement, large multicenter trials, and long-term tracking of skill retention and clinical transferability.

## Data Availability

The raw data supporting the conclusions of this article will be made available by the authors, without undue reservation.
