# Supplementary material for: Development and application of a standardized teaching assessment system for digital automated oral implantation planning skills: a randomized controlled pilot trial
Source: Front Oral Health. 2026 Sep 16;7:1892776. doi: 10.3389/froh.2026.1892776 (PMC13623744; doi:10.3389/froh.2026.1892776)
Supplement: Supplementary file 2 [file Table2.docx]

**Supplementary Material 2**

**Table 1 Inter-rater reliability (ICC) and clustering effect of raters for operation time**

|  | **ICC(2,1) [95%CI]** | **ICC(2,3) (average-measure reliability)** | **Main effect of rater p-value** |
| --- | --- | --- | --- |
| **Exp Group (n=20)** | 0.890 [.790, .950] | 0.960 | 0.967 |
| **Con Group (n=20)** | 0.907 [.820, .960] | 0.967 | 0.976 |
| **Total (n=40)** | 0.995 [.990, 1.000] | 0.998 | 0.998 |

*****Inter-rater reliability among the three raters was excellent, with ICC(2,1) ranging from 0.89 to 0.91 within each group. The main effect of raters yielded p > 0.05 for all subgroups, indicating no systematic measurement bias. One-way ANOVA was performed to test clustering effects across three raters (D1, D2, D3): F(2, 37) = 0.07, p = 0.936 (>0.05), demonstrating no significant clustering effect among raters.

**Table 2 Internal consistency reliability of the questionnaire (Cronbach's α)**

|  | **Total (n=40)** | **Exp group (n=20)** | **Con group (n=20)** |
| --- | --- | --- | --- |
| **System Usability Scale (SUS, 10 items）** | 0.811 | 0.747 | 0.741 |
| **DREEM Inventory**  **(5 domains)** | 0.885 | 0.715 | 0.745 |

**Note**: The Cronbach’s α coefficients of the total sample ranged from 0.7 to 0.9, indicating satisfactory internal consistency with this sample size.
