# Supplementary material for: Development and application of a standardized teaching assessment system for digital automated oral implantation planning skills: a randomized controlled pilot trial
Source: Front Oral Health. 2026 Sep 16;7:1892776. doi: 10.3389/froh.2026.1892776 (PMC13623744; doi:10.3389/froh.2026.1892776)
Supplement: Supplementary file 3 [file Table3.docx]

**Supplementary Material 3**

| **Outcome Measure** | **Shapiro-Wilk p (residual normality)** | **Levene’s p (homogeneity of variances)** | **Group × baseline interaction F (homogeneity of regression slopes)** | **p** |
| --- | --- | --- | --- | --- |
| **3D Deviation at the Implant Neck (mm)** | 0.823 | 0.999 | 0.10 | 0.753 |
| **3D Deviation at the Apical Region (mm)** | 0.969 | 0.724 | 0.24 | 0.628 |
| **Implant axial angle deviation (°)** | 0.383 | 0.949 | 0.09 | 0.762 |

*The p-values for the three prerequisite tests are all greater than 0.05, indicating that the residuals are normally distributed, the variance is homogeneous, and the regression slopes are homogeneous. The conditions for applying ANCOVA are met.
