# Supplementary material for: Development and application of a standardized teaching assessment system for digital automated oral implantation planning skills: a randomized controlled pilot trial
Source: Front Oral Health. 2026 Sep 16;7:1892776. doi: 10.3389/froh.2026.1892776 (PMC13623744; doi:10.3389/froh.2026.1892776)
Supplement: Supplementary file 4 [file Table4.docx]

**Supplementary Material 4**

The research team has developed a teaching software for oral implant evaluation, with the following specific content：

1. Homepage (**Figure 1**):

Figure 1: The homepage of Yake Bot

2. After logging in, enter the case management page, you can create patients, create plans, and import surgical plans. Click "Create Surgical Plan", select the "Oral Implant Evaluation" button, and the page will display as follows (**Figure 2**):

Figure 2: Select the "Oral Planting Evaluation" function in the software

3. Production plan. After opening the created dental implant planning, normally select the missing teeth positions of the patient, import the patient's CT data (add panoramic curve and panoramic points), import the patient's upper and lower jaw scanning data, complete the alignment of CT and scanning files, place the preoperative implant and abutment (place the implant by drawing arrows with the hand) (**Figure 3-4**).

Figure 3: Select the missing tooth position and match the model with the CT

Figure 4: Select the implant and abutment model, and set the ideal implant position

4. Implant Evaluation:

① Select the evaluation site (upper or lower jaw) based on the actual missing tooth location;

② Click the “Batch Import Postoperative Scans” button, select the postoperative scans, and import them in batches;

③ Perform intraoral scan registration. When the registration target is set to “All,” the system will complete the registration process for all postoperative and preoperative scans (**Figure 5**).

Figure 5: Match preoperative and postoperative scanning models

④ Complete the abutment registration. Once the scans have been registered, switch to the abutment registration function page. All abutments from the post-operative scans must be registered individually in sequence. Select three points on the corresponding abutment in the pre-operative scan on the left-hand side of the registration view, and select three points at the same locations on the corresponding abutment in the post-operative scan on the right-hand side to complete the three-point coarse registration of the abutment. If misalignment persists, a point cloud may be drawn over the misaligned areas to achieve precise registration (**Figure 6**).

Figure 6: Perform more precise matching based on the implant abutment

⑤ Click the ‘Show Labels’ button to display the implant position arrows and labels for the currently selected student, or to display the implant position arrows and labels for all students. The green arrows indicate the implant position for each student post-surgery (**Figure 7**).

Figure 7: After matching is completed, teacher can choose to display all students' implanting assignments

⑥ Once the allocation is complete, click ‘Display Chart’ to open the placement assessment chart page, which displays the allocation results for all students using various chart types. You can view the results by switching between the different chart types. Grading is also based on the chart to determine students’ marks. An example is shown below (**Figure 8**):

Figure 8:Click on 'Show Chart' to display data on students' oral implant assignments from multiple dimensions

⑦ Export evaluation results: Once all abutment registrations have been completed, click the ‘Export evaluation results’ button to export the post-operative evaluation results in .CSV format (**Figure 9**).

Figure 9:Click 'Export Assessment Results' to output all student assignment results in CSV format

⑧ Generate report: Select any scan, click the ‘Generate report’ button to generate a post-operative report based on the currently selected post-operative scan. (The registration results for the post-operative scan can be saved to the planning file) (**Figure 10**).

Figure 10: Click the 'Generate Report' button to generate postoperative reports for each student.
